# Evolving Landscape of Regenerative Therapies: Cell-Based and Cell-Free Approaches for Chronic Low Back Pain

**DOI:** 10.3390/jcm15135235

**Published:** 2026-07-04

**Authors:** Courtney E. Bartlett, Pareeshe Bansal, Siddhant Bhattacharya, Abhi Dhote, Bruna B. Nicoletto, Joana R. N. Lemos, Rahul Mittal

**Affiliations:** 1 College of Nursing, University of Arizona, Tucson, AZ 85721, USA; cebartlett@arizona.edu; 2 Diabetes Research Institute, University of Miami Miller School of Medicine, Miami, FL 33136, USA; pmbbansal@gmail.com (P.B.); redwoodant2010@hotmail.com (S.B.); abhidhote456@gmail.com (A.D.); bnicoletto@miami.edu (B.B.N.); joanalemos@miami.edu (J.R.N.L.); 3 Division of Endocrinology, Diabetes, and Metabolism, Department of Medicine, University of Miami Miller School of Medicine, Miami, FL 33136, USA

**Keywords:** chronic low back pain, intervertebral disc degeneration, mesenchymal stromal cells, platelet-rich plasma, platelet lysate, extracellular vesicles, secretome, cell-free therapy, regenerative medicine, discogenic pain

## Abstract

**Background:** Chronic low back pain (CLBP) is the leading cause of years lived with disability globally, affecting over 600 million individuals. Intervertebral disc degeneration (IVDD) is a principal structural contributor, yet conventional treatments, including pharmacotherapy, physical therapy, and surgical intervention, do not reverse the underlying degenerative pathology. Regenerative medicine has introduced a spectrum of biological therapies for IVDD, including cell-based mesenchymal stromal cell (MSC) therapy, platelet-derived products such as platelet-rich plasma (PRP) and platelet lysate, extracellular vesicle-based approaches using MSC-derived extracellular vesicles (EVs), and secretome-based therapies using MSC-derived secretomes. However, these approaches have largely been studied in isolation, without a unified framework to compare their respective advantages and limitations in CLBP secondary to IVDD. Accordingly, this narrative review aims to provide an integrated and comparative evaluation of these regenerative strategies within a single translational and clinical context. **Methods:** For this narrative review, PubMed, Scopus, and Web of Science were searched from January 2000 to January 2026 using terms combining regenerative modalities with intervertebral disc degeneration, and chronic low back pain. Randomized controlled trials (RCTs), prospective cohort studies, systematic reviews, and preclinical studies with translational relevance were included. **Results:** Intradiscal MSC therapy has demonstrated safety across multiple phase I–III trials, but two recent landmark RCTs (RESPINE and the Mesoblast phase III trial) failed to meet primary efficacy endpoints, highlighting the gap between preclinical promise and clinical outcomes. PRP has the largest clinical evidence base, with level II evidence supporting short- to medium-term pain relief for discogenic pain, although standardization remains a critical barrier. Platelet lysate, MSC-derived EVs, and MSC-derived secretomes show compelling preclinical data, including extracellular matrix restoration, anti-inflammatory modulation, and attenuation of nucleus pulposus cell apoptosis, but remain at early translational stages for spinal applications, with no completed RCTs. The hostile disc microenvironment (avascular, hypoxic, acidic, and nutrient-poor) poses unique challenges for all regenerative modalities, differing fundamentally from other musculoskeletal applications. **Conclusions:** The studies included in this narrative review suggest that no single regenerative modality has yet shown consistent and unequivocal efficacy for CLBP secondary to IVDD across clinical trials. Cell-free approaches offer manufacturing, scalability, and safety advantages over cell-based therapies, but lack clinical validation. Future progress requires standardized preparation protocols, disc-specific delivery systems, patient phenotyping strategies, and rigorously designed comparative clinical trials. This narrative review provides a framework for researchers and clinicians to evaluate these therapies in context rather than isolation.

## 1. Introduction

Low back pain (LBP) remains the leading cause of disability worldwide, affecting an estimated 619 million individuals in 2020, with projections suggesting approximately 843 million cases by 2050 as the populations age [1,2,3,4,5]. Chronic low back pain (CLBP), defined as pain persisting beyond 12 weeks, accounts for the majority of direct healthcare expenditure and indirect costs attributable to lost productivity, with estimated annual costs exceeding $100 billion in the United States alone [6,7,8,9]. Despite this extraordinary burden, the therapeutic landscape remains dominated by interventions that manage symptoms, such as analgesics, physical therapy, spinal injections, and surgical procedures including fusion and disc replacement, rather than addressing the underlying degenerative pathology [10,11,12,13].

Intervertebral disc degeneration (IVDD) is recognized as one of the most prevalent structural contributors to CLBP, though the relationship between radiographic degeneration and clinical symptoms is complex and non-linear [14,15,16,17]. The intervertebral disc is the largest avascular structure in the human body, consisting of a proteoglycan-rich, hydrated nucleus pulposus (NP) enclosed by the collagen-rich annulus fibrosus (AF), with cartilaginous endplates (CEPs) providing the primary route for nutrient diffusion [18,19,20,21]. Degeneration involves a cascade of interrelated pathological processes. These include loss of notochordal cells and a progressive decline in function and number of nucleus pulposus (NP) cells [22,23,24,25]. It also involves extracellular matrix (ECM) catabolism with proteoglycan depletion and collagen remodeling, endplate calcification that reduces nutrient transport, neovascularization and neoinnervation of the annulus fibrosus (AF), and a self-perpetuating inflammatory milieu dominated by interleukin-1β (IL-1β), tumor necrosis factor-α (TNF-α), and matrix metalloproteinases (MMPs) [26,27,28,29,30,31].

The regenerative medicine field has produced a growing spectrum of biological therapies intended to counteract these degenerative processes. These include cell-based approaches, principally intradiscal injections of mesenchymal stromal cells (MSCs); platelet-derived products such as platelet-rich plasma (PRP) and platelet lysate (PL); extracellular vesicle-based approaches involving MSC-derived extracellular vesicles (EVs); and secretome-based therapies comprising MSC-derived secretomes or conditioned media (CM). Each modality operates through distinct but overlapping mechanisms: MSCs through paracrine signaling and, theoretically, differentiation into NP-like cells; platelet-derived products through concentrated growth factor delivery; and MSC-derived extracellular vesicles and secretomes through targeted intercellular signaling molecules including miRNAs, cytokines, and trophic factors [32,33,34,35,36].

However, the literature on these regenerative therapies for CLBP has developed in isolated silos. Existing reviews address MSC therapy for disc degeneration [37,38,39], PRP for spinal disorders [40,41,42], or EVs for IVDD [43,44,45] as separate topics. To the best of our knowledge, no published review has integrated cell-based MSC therapy, platelet-derived therapies (PRP and platelet lysate), and MSC-derived cell-free therapies (EVs and secretomes) into a single comparative framework for CLBP. This gap is clinically significant because treatment selection requires understanding the relative advantages and limitations of each approach in the context of the unique challenges posed by the disc microenvironment. This environment differs fundamentally from the vascularized tissues (skin, joint, bone) where these therapies have been more extensively studied.

This narrative review addresses the current lack of an integrated comparative evaluation of regenerative therapies for CLBP secondary to IVDD, an important limitation in the existing literature with implications for both clinical practice and translational research. As biological and regenerative interventions gain increasing attention in spine medicine, a comprehensive framework is needed to critically assess the relative advantages, limitations, and stages of clinical development of the available therapeutic modalities. In this review, we examine the biological rationale for regenerative therapy in IVDD, evaluate each modality by considering both preclinical evidence and clinical trial data, as well as providing comparative analyses using structured summary tables. We also discuss key translational challenges relevant to intradiscal application, including delivery strategies, manufacturing considerations, and regulatory issues. By integrating the current evidence across cell-based and cell-free approaches, this review aims to clarify the current state of the field, highlight existing knowledge gaps, and identify priorities for future research and clinical translation in the management of IVDD-associated CLBP.

This narrative review focuses primarily on MSC-based therapies as the representative cell-based modality, given their relatively advanced stage of clinical development and the availability of randomized and late-phase clinical trial data. Other cellular approaches, including bone marrow aspirate, bone marrow concentrate, and discogenic cell therapies, are recognized as relevant but are not comprehensively addressed, as their inclusion would extend beyond the scope of the present comparative framework.

The scope of this review is intentionally focused on the comparative evaluation of MSC-based therapies and cell-free regenerative approaches, including platelet-rich plasma, platelet lysate, MSC-derived extracellular vesicles, and MSC-derived secretomes, for chronic low back pain associated with IVDD. Although other regenerative biologic strategies, such as bone marrow concentrate, bone marrow aspirate, and disc-derived cell therapies, are recognized as important areas of investigation, a comprehensive review of all biologic interventions would extend beyond the intended scope of this article. These therapies are therefore discussed only briefly where relevant to provide context regarding the broader regenerative medicine landscape.

Unlike previous reviews that focus on individual regenerative modalities, the present review provides a comparative evaluation of cell-based and cell-free approaches within a single translational framework. Such a comparison is increasingly important as multiple regenerative strategies progress through different stages of clinical development and may ultimately compete for similar patient populations.

## 2. Methods

### 2.1. Study Design

This study was conducted as a narrative review incorporating a structured literature search to support a comprehensive and transparent synthesis of the field. Although predefined databases, search terms, and eligibility criteria were applied, this review does not conform to formal systematic or scoping review methodologies, as it does not follow PRISMA guidelines, include a study selection flow diagram, or incorporate a formal risk-of-bias assessment. Instead, the objective was to provide an interpretive and comparative evaluation of regenerative therapies for CLBP secondary to IVDD, integrating mechanistic insights with available preclinical and clinical evidence.

### 2.2. Literature Search Strategy

For this narrative review, a literature search was performed using PubMed, Scopus, and Web of Science to identify relevant studies published between January 2000 and January 2026. Search strategies incorporated controlled vocabulary (such as Medical Subject Headings [MeSH]) and free-text terms, including but not limited to, “chronic low back pain”, “intervertebral disc degeneration”, “discogenic pain”, “mesenchymal stromal cells”, “mesenchymal stem cells”, “platelet-rich plasma”, “platelet lysate”, “extracellular vesicles”, “secretome”, and “regenerative medicine”. Boolean operators (AND, OR) were applied to optimize sensitivity and specificity of retrieval.

To further enhance reproducibility, the final search was conducted in January 2026, and the search strategy was applied across PubMed, Scopus, and Web of Science using database-adapted combinations of the above terms. Searches were limited to English-language articles. As this was a narrative review rather than a systematic review, formal PRISMA-based record tracking was not performed. However, studies were prioritized for inclusion based on relevance to IVDD-associated CLBP, level of evidence, clinical or translational significance, methodological rigor, and representation of major therapeutic modalities.

### 2.3. Eligibility Criteria

Studies were considered eligible if they met the following criteria:

(i) investigated regenerative or biologic therapies targeting IVDD or discogenic CLBP;
(ii) included randomized controlled trials, prospective or retrospective observational studies, systematic reviews, or preclinical investigations with translational relevance; and
(iii) reported outcomes related to biological mechanisms, structural disc changes, or clinical endpoints (e.g., pain or functional measures).

Exclusion criteria comprised:

(i) studies not specifically addressing discogenic pathology;
(ii) articles lacking relevance to regenerative or biologic interventions;
(iii) editorials, commentaries, or opinion pieces without primary or synthesized data.

Given the challenges associated with definitively establishing a discogenic source of pain, studies were included if they evaluated patients with chronic low back pain in the presence of imaging findings consistent with intervertebral disc degeneration. It is acknowledged that most clinical studies do not employ confirmatory diagnostic techniques, such as provocative discography, and therefore the classification of discogenic pain in the included literature reflects a pragmatic, study-level definition rather than a uniformly validated diagnosis.

To avoid conceptual ambiguity, regenerative modalities in this review are classified as cell-based therapies, platelet-derived products, acellular soluble-factor products, and vesicle-based products. Cell-based therapies refer to interventions containing viable nucleated cells, such as mesenchymal stromal cells. Platelet-derived products include platelet-rich plasma and platelet lysate. Acellular soluble-factor products refer to formulations dominated by soluble secreted proteins, cytokines, and growth factors without viable cells. Vesicle-based products include extracellular vesicles and exosomes, which are acellular but contain membrane-bound subcellular structures capable of delivering bioactive molecular cargo.

### 2.4. Study Selection and Data Synthesis

Titles and abstracts were screened for relevance, followed by full-text evaluation of potentially eligible studies. Given the substantial heterogeneity in study design, intervention protocols, outcome measures, and follow-up durations, quantitative synthesis or meta-analysis was not undertaken. Instead, data were synthesized qualitatively with emphasis on (i) underlying biological mechanisms; (ii) preclinical efficacy; (iii) clinical safety and effectiveness; and (iv) translational, manufacturing, and regulatory considerations. A comparative framework was applied to evaluate the relative advantages, limitations, and stages of development of each therapeutic modality. Where appropriate, representative landmark clinical trials were selectively highlighted to represent the highest level of available evidence for each therapeutic modality, rather than to provide an exhaustive summary of the literature. Given the heterogeneity of study designs, interventions, and outcome measures across the included literature, findings are presented as a qualitative narrative synthesis rather than a quantitatively pooled or systematically graded analysis of evidence. A structured comparative framework was applied to evaluate each modality across predefined domains, including mechanism of action, level of clinical development, standardization and manufacturing considerations, interaction with the disc microenvironment, and key translational and regulatory challenges.

Study selection decisions were made through discussion among the authors, with emphasis on including studies most relevant to the review’s comparative translational framework. Disagreements regarding study relevance or interpretation were resolved by consensus. Landmark studies were defined as randomized or controlled clinical trials, systematic reviews, or highly cited preclinical/translational studies that substantially influenced understanding of regenerative therapies for IVDD-associated CLBP.

As this is a narrative review rather than a systematic review or meta-analysis, comparisons across therapeutic modalities were interpreted cautiously. The included studies differed substantially in design, patient populations, treatment protocols, endpoints, and follow-up duration. Therefore, comparative statements are intended to provide a qualitative overview of relative clinical maturity, translational status, and evidence gaps rather than definitive conclusions regarding comparative efficacy.

## 3. The Degenerative Disc Microenvironment: Implications for Regenerative Therapy

### 3.1. Structural and Cellular Degeneration

The intervertebral disc undergoes progressive structural and cellular changes with aging that are accelerated by mechanical overload, genetic predisposition, metabolic dysfunction, and lifestyle factors including smoking and obesity [18,46]. The NP transitions from a gelatinous, proteoglycan-rich tissue maintained by notochordal cells in youth to a more fibrotic, dehydrated structure dominated by smaller chondrocyte-like cells in adulthood. This cellular transition is accompanied by a decline in aggrecan and type II collagen synthesis, increased production of type I collagen and fibronectin fragments, and progressive loss of the glycosaminoglycan content responsible for osmotic water retention [47,48]. The AF develops radial tears and fissures that compromise mechanical containment and provide pathways for neoinnervation and neovascularization, the structural basis for discogenic pain [49].

### 3.2. The Hostile Disc Microenvironment

The disc microenvironment presents unique challenges for any regenerative therapy. Unlike vascularized tissues such as skin or joint synovium, the NP is avascular, with nutrient supply dependent on passive diffusion through the CEPs [50]. Oxygen tension in the central NP is extremely low (1–5% O_2_), and pH ranges from 6.5 to 7.1 under normal conditions, falling further to 6.0–6.5 in degenerated discs [51]. Glucose concentrations are approximately one-fifth of serum levels [52]. These conditions have direct implications for regenerative therapy. Transplanted cells face substantial challenges related to survival, metabolic activity, and functional persistence within the disc. At the same time, acellular therapies are not exempt from these constraints, as growth factors and extracellular vesicle-associated cargo may be susceptible to degradation, altered diffusion, and reduced bioactivity under acidic, hypoxic, and nutrient-depleted conditions. Accordingly, the hostile disc environment does not selectively disadvantage one modality but instead imposes distinct limitations on both cell-based and cell-free approaches.

Importantly, the efficacy of cell-free therapies is dependent on the presence of viable and functionally responsive endogenous disc cells [53,54]. In degenerated discs, reductions in cell density, increased cellular senescence, and altered receptor expression may limit the capacity of resident cells to respond to exogenous bioactive signals [55,56]. This introduces an additional layer of constraint, as therapeutic effectiveness depends not only on the stability of delivered factors but also on the biological competence of the target cell population [57,58].

**Figure 1:**
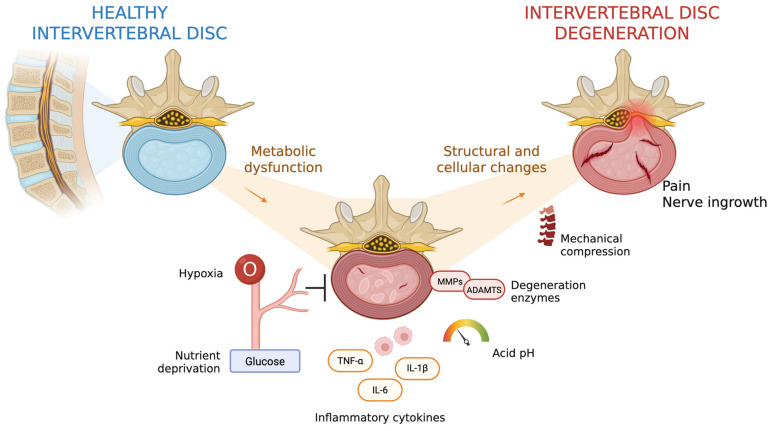
Pathophysiological mechanisms of intervertebral disc degeneration. A healthy intervertebral disc maintains extracellular matrix homeostasis within a hypoxic but metabolically balanced microenvironment. Degeneration is initiated by metabolic dysfunction, including hypoxia, reduced nutrient availability, and acidification, which promote inflammatory cytokine production (TNF-α, IL-1β, and IL-6) and upregulation of matrix-degrading enzymes (MMPs and ADAMTS). These changes drive extracellular matrix degradation, structural disruption, and mechanical instability, facilitating nerve ingrowth and pain generation. The resulting microenvironment establishes a self-perpetuating degenerative cycle that underlies discogenic chronic low back pain. Created with Biorender.com.

These factors collectively highlight that the disc microenvironment represents a shared barrier to regenerative therapies, with modality-specific vulnerabilities rather than inherent advantages of one approach over another. This may partly explain why preclinical efficacy in animal models has not consistently translated to clinical benefit in human trials [37,59] (Figure 1).

### 3.3. The Inflammatory and Catabolic Cascade

IVDD is driven by a self-perpetuating inflammatory cycle. Degenerated NP cells and infiltrating macrophages produce elevated levels of pro-inflammatory cytokines, primarily IL-1β, TNF-α, and IL-6, which upregulate MMPs (MMP-1, -3, -13) and ADAMTS (a disintegrin and metalloproteinase with thrombospondin motifs) enzymes responsible for ECM degradation [28,60]. These inflammatory mediators simultaneously suppress anabolic gene expression (aggrecan, type II collagen, SOX9), promote NP cell apoptosis and senescence, and sensitize AF nociceptors [61,62]. The catabolic–anabolic imbalance provides the primary therapeutic target for regenerative interventions; therefore, effective therapies must deliver pro-anabolic signals and modulate the inflammatory milieu to prevent degradation of any newly synthesized matrix [29,63].

### 3.4. Multi-Source Pain Generation

It is important to recognize that CLBP is frequently multifactorial, not arising solely from disc degeneration. Facet joint arthropathy, sacroiliac joint dysfunction, paraspinal muscle deconditioning and fatty infiltration, and ligamentous instability each contribute independently or in combination [64,65]. One large case series of CLBP patients treated with PRP found that 82% had pathological changes at two or more anatomical sites [40]. This multifactorial nature complicates clinical trial design for disc-specific regenerative therapies and may partly account for high placebo response rates observed in recent RCTs. An effective regenerative strategy may ultimately need to address multiple pain generators, not only the disc [66].

## 4. Cell-Based Therapy: Intradiscal Mesenchymal Stromal Cell Injection

### 4.1. Biological Rationale

MSCs derived from bone marrow (BM-MSCs), adipose tissue (AD-MSCs), or umbilical cord tissue (UC-MSCs) have attracted attention for IVDD therapy based on two proposed mechanisms: differentiation into NP-like cells capable of restoring ECM synthesis, and paracrine signaling that modulates inflammation, suppresses apoptosis, and stimulates endogenous repair [32,67]. Preclinical studies suggest that MSCs co-cultured with NP cells upregulate aggrecan and type II collagen expression, reduce MMP-3 and IL-1β levels, and adopt an NP-like phenotype characterized by expression of cytokeratin-19 and brachyury [68,69]. In animal models of IVDD, intradiscal MSC injection has been associated with improvements in disc height, MRI signal intensity (T2-weighted), and histological scoring [37,70,71].

### 4.2. Clinical Evidence

Translation to clinical practice has been sobering. A 2025 PRISMA-compliant systematic review identified 13 clinical studies (2011–2025) enrolling 1299 patients, comprising 5 RCTs and 8 prospective or pilot studies [37]. Cell sources included BM-MSCs (most common), AD-MSCs, and UC-MSCs, with doses ranging from 2 × 10^6^ to 4 × 10^7^ cells per disc and follow-up from 12 months to 6 years. While individual studies reported improvements in Visual Analog Scale (VAS) pain scores and Oswestry Disability Index (ODI), landmark RCTs have tempered enthusiasm. To provide an overview of the current state of clinical translation, selected randomized and controlled trials are described below, with a focus on studies considered to have relatively robust design, larger sample sizes, or more advanced clinical development.

The RESPINE Trial (2024): This multicenter, double-blind, placebo-controlled trial randomized 114 patients with single-level CLBP to receive intradiscal injections of 20 million allogeneic BM-MSCs or sham injection. At 12 months, the primary composite endpoint was not met (74% responders in the MSC group vs. 69% in the placebo; *p* = 0.77). MRI disc fluid content showed no significant between-group difference. The trial was methodologically robust and represents the first adequately powered, double-blind, sham-controlled study of allogeneic MSCs for CLBP [72].

The Mesoblast Phase III Trial (2025): This prospective, randomized, double-blind trial evaluated 6 million allogeneic mesenchymal precursor cells (MPCs) with or without hyaluronic acid (HA) versus saline in 404 patients over 36 months. The primary composite endpoint was not met. Although MPC+HA was associated with pain reduction in some analyses, including a subgroup of patients with CLBP duration less than 68 months, these findings should be interpreted as exploratory unless prespecified and appropriately adjusted for multiplicity [73].

It should be noted that the study population included patients with chronic low back pain and imaging evidence of degenerative disc disease, which does not necessarily confirm a discogenic pain source. Accordingly, interpretation of clinical outcomes should consider the potential contribution of non-discogenic pain generators.

The DREAM Study (2025): This phase IIB, double-blind RCT of autologous BM-MSCs for multilevel IVDD in 52 patients found significant structural improvement (increased disc height index) but no superior clinical outcomes compared to sham at 6 months [74].

For trials that did not meet their primary endpoint, secondary or subgroup findings should be interpreted cautiously. Unless such analyses were prespecified and appropriately adjusted for multiplicity, favorable secondary or subgroup outcomes should be considered exploratory and hypothesis-generating rather than confirmatory evidence of efficacy.

### 4.3. Challenges and Limitations

Several factors may explain the clinical-preclinical disconnect. First, the biochemically adverse environment compromises transplanted cell survival. Studies suggest that fewer than 10% of injected MSCs remain viable at 48 h post injection in degenerated discs [59]. Second, the disc is largely immune-privileged but not completely immunologically inert—allogeneic cells may trigger low-grade immune responses that limit their paracrine activity [75]. Third, high placebo response rates in CLBP trials (approaching 70% in RESPINE) reduce statistical power to detect treatment effects. Fourth, heterogeneity in cell source, dose, passage number, culture conditions, and delivery vehicle across trials prevents meaningful cross-study comparison [37]. Fifth, the selection of appropriate patient populations—specifically, identifying patients whose pain is predominantly discogenic and at a stage of degeneration amenable to biological repair—remains a critical unsolved challenge [73].

It should be noted that additional cellular approaches have been investigated for intervertebral disc regeneration, including bone marrow aspirate, bone marrow concentrate, and discogenic cell therapies [76,77,78]. Disc-derived allogeneic cell products have demonstrated promising results in recent clinical studies, including randomized trials evaluating their safety and efficacy in patients with chronic low back pain associated with disc degeneration [76,79]. These approaches differ from MSC-based therapies in terms of biological rationale, manufacturing processes, and stage of clinical development, and are therefore not directly comparable within the framework of MSC-based versus cell-free strategies emphasized in this review. Notably, the DiscGenics program represents a leading example of this class of therapies, with randomized clinical data demonstrating clinically meaningful improvements in pain and functional outcomes [76].

## 5. Platelet-Rich Plasma for Chronic Low Back Pain

### 5.1. Biological Rationale

PRP is an autologous blood concentrate containing supraphysiological concentrations (typically 3–7-fold above baseline) of platelets, which upon activation release a cocktail of growth factors including platelet-derived growth factor (PDGF), transforming growth factor-β (TGF-β), vascular endothelial growth factor (VEGF), insulin-like growth factor-1 (IGF-1), and basic fibroblast growth factor (bFGF) [80]. In vitro, PRP release stimulates NP cell proliferation, upregulates aggrecan and type II collagen synthesis, and suppresses expression of IL-1β, MMP-3, and nerve growth factor (NGF) [81,82]. Animal studies using needle puncture IVDD models in rabbits, rats, and pigs suggest that intradiscal PRP injections can restore disc height, improve MRI T2 signal, and attenuate histological degeneration [41,83,84].

### 5.2. Clinical Evidence

PRP has the largest clinical evidence base among regenerative therapies for CLBP. Importantly, the clinical evidence for PRP should be interpreted with caution, as studies include heterogeneous injection targets, including intradiscal, facet joint, epidural, and paraspinal applications. Therefore, evidence supporting PRP for CLBP more broadly should not be directly equated with evidence for intradiscal treatment of IVDD or structural disc regeneration. A 2023 systematic review graded the evidence as level II quality supporting PRP use in CLBP, though noting the need for large-scale multicenter RCTs [40]. A 2024 network meta-analysis of RCTs comparing PRP to various controls (corticosteroids, saline, radiofrequency ablation) found that PRP and radiofrequency were comparably effective for pain reduction, but PRP showed advantages in disability improvement at 6 months or more of follow-up [85]. Clinical applications span multiple spinal pain generators: intradiscal injection for discogenic pain, intra-articular injection for facet joint arthropathy, epidural injection for radicular pain, and paraspinal muscle injection for myofascial pain and multifidus atrophy [40,86]. This versatility across multiple anatomical targets is a distinctive advantage given the multifactorial nature of CLBP [65].

In contrast to cell-based therapies, the PRP literature comprises a broader but more heterogeneous body of clinical studies, including multiple randomized trials with variable methodologies. As a result, evidence is often synthesized at the level of systematic reviews and meta-analyses rather than anchored to a small number of large, definitive trials.

### 5.3. Limitations

Critical limitations include the absence of standardized preparation protocols. PRP products vary widely in platelet concentration, leukocyte content (leukocyte-rich vs. leukocyte-poor), activation method (thrombin, calcium chloride, freeze–thaw), and volume delivered [87]. These variations directly affect growth factor profiles and may account for inconsistent clinical outcomes across studies. Furthermore, as an autologous product, PRP quality is dependent on the patient’s own platelet function and count, which may be compromised by age, comorbidities, or medication use (e.g., NSAIDs, anticoagulants) [88]. The short half-life of released growth factors in the disc environment raises questions about the durability of single-injection protocols. Evidence for true biological disc regeneration (as opposed to anti-inflammatory and analgesic effects) in humans remains lacking, as no clinical study has suggested sustained MRI improvement attributable to PRP [40,41]. Thus, the currently available clinical evidence for PRP is best interpreted as supporting possible analgesic and functional improvement in selected patients, rather than confirmed biological regeneration of the intervertebral disc.

## 6. Platelet Lysate: An Emerging Alternative to PRP

### 6.1. Biological Rationale and Distinction from PRP

Platelet lysate (PL) is produced by freeze–thaw cycling or sonication of platelet concentrates, resulting in complete lysis of platelet membranes and release of the total intracellular growth factor content, followed by removal of cell debris by centrifugation or filtration [89]. Unlike PRP, which requires in situ activation to release growth factors from intact platelet α-granules, PL delivers a pre-released, more complete growth factor complement including higher and more reproducible concentrations of PDGF, TGF-β, VEGF, IGF-1, and hepatocyte growth factor (HGF) [90,91]. PL can be prepared from pooled allogeneic platelets, enabling standardized, quality-controlled batch production—a significant advantage over autologous PRP [92].

### 6.2. Preclinical Evidence for Disc Regeneration

In vitro studies have suggested that PL stimulates NP cell and AF cell proliferation, enhances proteoglycan and type II collagen synthesis, and suppresses catabolic gene expression under inflammatory conditions [93,94]. A seminal 2014 study first identified EVs as effectors within PL, demonstrating that PL-derived EVs promoted dose-dependent proliferation and migration of bone marrow stromal cells [34]. This finding suggests that PL activity extends beyond soluble growth factors to include EV-mediated signaling. In animal models of IVDD, Limited in vitro evidence suggests that platelet lysate may support nucleus pulposus cell viability and regenerative activity in disc-related models [93].

### 6.3. Clinical Evidence and Gaps

Clinical evidence for PL in spinal applications is extremely limited. While PL has been studied in orthopedic contexts (notably knee osteoarthritis, where one RCT comparing PL to PRP found comparable outcomes [95]), no completed RCT has evaluated intradiscal PL injection for CLBP. The advantages of standardized, allogeneic PL production and more complete growth factor release make this a promising candidate for disc regeneration, however clinical validation is urgently needed. The ability to produce PL under GMP conditions with defined potency specifications addresses a key limitation of autologous PRP—donor-dependent variability—and may be particularly relevant for CLBP patients who are typically older and may have suboptimal platelet function [89,92].

## 7. MSC-Derived EVs for Intervertebral Disc Regeneration

### 7.1. Biological Rationale

The recognition that MSC therapeutic effects are primarily paracrine rather than dependent on engraftment and differentiation has driven interest in MSC-derived EVs as cell-free alternatives [33,35]. Exosomes are nanoscale EVs (30–150 nm) of endosomal origin that carry a cargo of miRNAs, mRNAs, proteins, and lipids reflecting the parent cell’s biological state [96]. In the context of IVDD, MSC-derived EVs may modulate each of the key pathological processes: attenuating inflammation (via suppression of the NF-κB and NLRP3 inflammasome pathways), inhibiting NP cell apoptosis (via PI3K/AKT and MAPK signaling), promoting ECM synthesis (via delivery of miR-142-3p, miR-199a, and other regulatory miRNAs), and reducing oxidative stress [43,44,45,97].

### 7.2. Preclinical Evidence

Preclinical studies have proliferated rapidly. BM-MSC-derived EVs promote NP cell proliferation and healthier ECM production. BM-MSC-derived extracellular vesicles have been shown in preclinical IVDD models to promote nucleus pulposus cell survival, reduce inflammatory and catabolic signaling, and support extracellular matrix homeostasis [98]. ADSC-derived EVs have been associated with angiogenesis inhibition (beneficial in the disc context, where neovascularization contributes to pain) and anti-inflammatory effects [45]. UC-MSC-derived exosomes have been shown to improve nucleus pulposus cell viability and reduce pyroptosis through miR-26a-5p/METTL14/NLRP3 signaling in IVDD models [99]. Nucleus pulposus cell-derived EVs themselves may induce BM-MSCs to differentiate toward an NP-like phenotype, suggesting endogenous vesicle-mediated repair pathways that therapeutic EVs might amplify [43]. Engineering strategies, including surface modification, hypoxic preconditioning, and loading with specific miRNA cargo, have further enhanced exosome therapeutic potencies in preclinical models [100].

While the majority of studies have focused on MSC-derived EVs, it is important to recognize that EVs can be derived from multiple cell types, including native intervertebral disc cells. Emerging evidence suggests that disc cell-derived EVs may exhibit greater tissue specificity and enhanced anabolic activity, potentially reflecting adaptation to the disc microenvironment. For example, it has been shown that EVs derived from nucleus pulposus cells can promote extracellular matrix synthesis and modulate degenerative pathways in a manner that may be more closely aligned with native disc biology [101]. These findings indicate that the cellular source of EVs represents a critical determinant of therapeutic function and should be considered in the development of EV-based regenerative strategies.

### 7.3. Translational Challenges

Despite compelling preclinical data, no clinical trial has evaluated MSC-derived EVs for CLBP or IVDD in humans. Critical translational barriers include: isolation and purification methods that remain non-standardized (ultracentrifugation, tangential flow filtration, size exclusion chromatography each yielding different purity and yield profiles); batch-to-batch variability in cargo composition; the absence of validated potency assays specific to disc regeneration; uncertain pharmacokinetics in the avascular, acidic disc environment; and an evolving regulatory landscape in which exosome products occupy an undefined space between drugs and biologics [43,102]. The International Society for Extracellular Vesicles (ISEV) MISEV2023 guidelines provide a framework for characterization, but standardized manufacturing for clinical application remains a work in progress [103].

## 8. MSC-Derived Secretomes and Conditioned Media

### 8.1. Definition and Distinction from Exosomes

The MSC secretome encompasses the totality of molecules secreted by MSCs into their culture medium, including soluble proteins (growth factors, cytokines, chemokines), EVs (exosomes and microvesicles), and small molecule metabolites [104]. Conditioned media (CM) represents the simplest form of secretome harvest: culture medium collected after a defined incubation period. The secretome differs from isolated EV preparations in that it contains the full spectrum of paracrine factors rather than only the vesicle-packaged cargo. This broader molecular composition may provide synergistic therapeutic effects through multiple simultaneous signaling pathways, although introducing a greater batch variability and characterization challenges [105].

### 8.2. Preclinical Evidence for Disc Regeneration

A proteomic analysis of extracellular vesicles derived from human IVD cells identified over 1800 proteins, underscoring the molecular complexity of paracrine signaling in the disc [106]. MSC-CM has been suggested to exert anti-inflammatory, anti-apoptotic, and matrix-protective effects in vitro using NP cell culture models under degenerative conditions [107]. One study evaluating the secretome of MSCs exposed to healthy, traumatic, and degenerative disc environments found that the secretome composition was dynamically modulated by the disc milieu, suggesting that preconditioning strategies may be used to tailor secretome content for specific disc pathologies [33]. Compared with isolated exosome preparations, the complete secretome may offer advantages in disc applications where multiple degenerative pathways (inflammation, apoptosis, matrix catabolism, oxidative stress) operate simultaneously and may benefit from multi-target intervention [105].

### 8.3. Clinical Status and Gaps

As with EVs, no clinical trial has evaluated MSC-derived secretomes for CLBP or IVDD. The secretome faces similar translational challenges regarding standardization, potency assays, and regulatory classification. An additional challenge is the larger volume and potentially less defined molecular composition compared to purified exosome preparations, which complicates dose determination and quality control. However, the simpler manufacturing process (collection and filtration of conditioned media vs. multi-step exosome isolation) may offer practical advantages for scalable production [104,105].

## 9. Comparative Analysis of Cell-Based, Platelet-Derived, Extracellular Vesicle-Based, and Secretome-Based Approaches

The comparative distinction among these modalities is shaped by their mechanism of action, degree of clinical validation, and translational feasibility. As discussed in Section 3, the degenerative disc microenvironment imposes major constraints on all regenerative therapies. For MSC-based therapies, these constraints primarily affect cell survival, engraftment, and paracrine activity. For platelet-derived, vesicle-based, and secretome-based approaches, the same environment may affect molecular stability, diffusion, retention, and responsiveness of resident disc cells.

Thus, although non-cellular approaches may offer advantages related to manufacturing, storage, and avoidance of viability-dependent limitations, they do not eliminate the major biological and delivery barriers associated with intradiscal therapy. At present, no modality has consistently demonstrated reproducible structural regeneration or clear clinical superiority for IVDD-associated CLBP.

**Figure 2:**
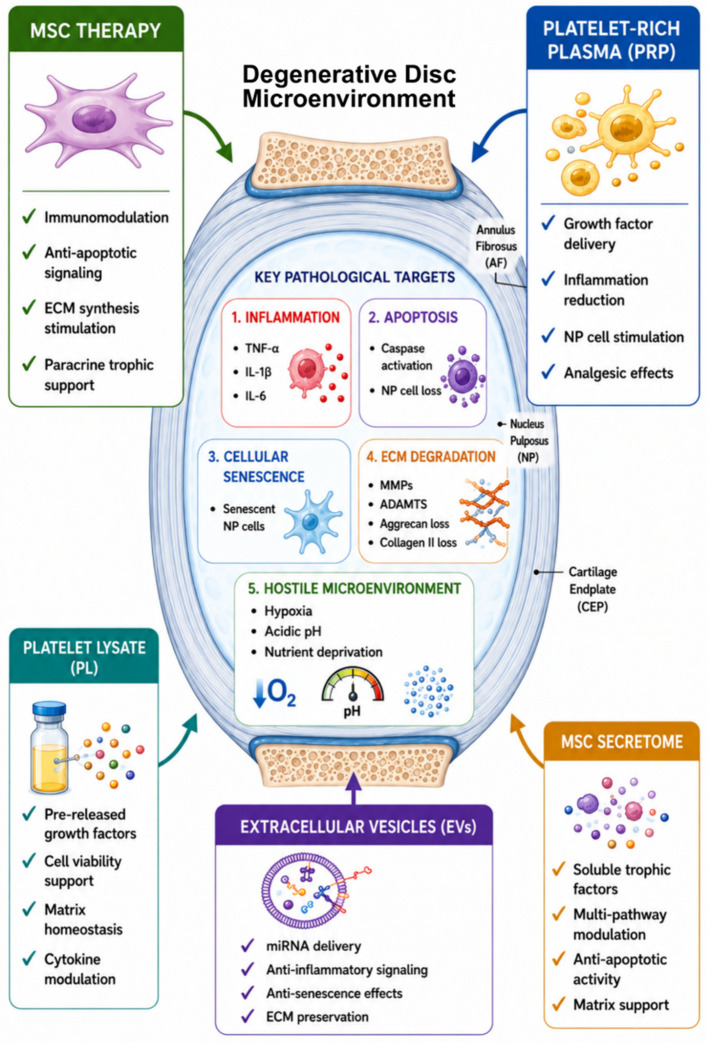
Proposed therapeutic targets of regenerative modalities within the degenerative disc microenvironment. MSC-based therapies act primarily through paracrine signaling, immunomodulation, anti-apoptotic effects, and potential stimulation of extracellular matrix synthesis, although their activity depends on cell survival within the hypoxic, acidic, and nutrient-poor disc environment. Platelet-rich plasma delivers platelet-derived growth factors that may reduce inflammation, support nucleus pulposus cell activity, and provide analgesic effects, but current clinical evidence does not establish consistent structural disc regeneration. Platelet lysate provides pre-released platelet-derived growth factors and bioactive mediators, with potential effects on cell viability, matrix homeostasis, and inflammatory modulation. Extracellular vesicle-based therapies deliver regulatory proteins, lipids, and nucleic acids that may modulate inflammation, apoptosis, senescence, and extracellular matrix metabolism. Secretome-based therapies provide a broader mixture of soluble factors and vesicular components that may target multiple degenerative pathways simultaneously. The mechanisms shown in this figure should be interpreted as proposed therapeutic pathways, with some supported by clinical evidence and others remaining primarily preclinical or hypothetical. Created in BioRender. Mittal, R. (2026) https://BioRender.com/q98omco (accessed on 17 June 2026).

Table 1 presents a structured comparison of the regenerative modalities across key parameters relevant to CLBP management. Notably, PRP occupies an intermediate position within this framework, as its therapeutic effects are mediated through the activation and degranulation of platelet components rather than through viable nucleated cells or purely acellular molecular delivery. Figure 2 shows the principal pathological targets within the degenerative disc microenvironment and the proposed mechanisms through which MSC-based and cell-free regenerative therapies may modulate these pathways. Importantly, the mechanisms depicted in Figure 2 are not supported by equivalent levels of evidence across modalities. While some effects of PRP and MSC-based therapies are supported by clinical studies, many mechanisms shown for platelet lysate, extracellular vesicle-based therapies, and secretome-based therapies remain primarily preclinical or hypothetical in the context of IVDD-associated chronic low back pain. Therefore, the figure should be interpreted as a conceptual summary of proposed therapeutic mechanisms rather than evidence of clinically established efficacy for all listed effects.

Platelet-rich plasma is categorized separately from both cell-based and acellular therapies, as it contains anucleate but metabolically active platelet components and may include leukocytes depending on preparation methods. As such, it does not meet the strict definition of a cell-free formulation, but also differs fundamentally from nucleated cell-based therapies.

To enable a more systematic and domain-specific evaluation, the following sections examine cell-based and cell-free modalities across key dimensions, including mechanism of action, interaction with the disc microenvironment, clinical maturity, and translational feasibility.

### 9.1. Mechanism of Action and Biological Activity

Across these domains, a primary distinction lies in the mechanisms by which therapeutic effects are mediated. Cell-based therapies, particularly MSCs, act predominantly through paracrine signaling, involving the secretion of cytokines, growth factors, and extracellular vesicles that regulate inflammatory pathways, attenuate apoptosis, and promote extracellular matrix synthesis [108,109,110]. In addition, these cells exhibit limited capacity for phenotypic adaptation toward nucleus pulposus-like lineages, contributing to tissue-specific reparative processes. This combination of secretory activity and potential lineage plasticity enables a dynamic interaction with the local microenvironment.

In contrast, cell-free approaches, including platelet-rich plasma, platelet lysate, and mesenchymal stromal cell-derived extracellular vesicles and secretomes, function through the direct delivery of bioactive molecular components without reliance on viable cells. These therapies provide a defined repertoire of signaling mediators, such as platelet-derived growth factor, transforming growth factor beta, and regulatory microRNAs, which act on resident disc cells to modulate anabolic and catabolic pathways. EV-based formulations further enable intercellular communication through the transfer of nucleic acids and proteins, influencing gene expression and cellular behavior at a post-transcriptional level.

The distinction between these modalities reflects a fundamental trade-off between biological adaptability and mechanistic control. Cell-based therapies offer the potential for sustained, environmentally responsive signaling, contingent upon cell survival and functional persistence within the disc. In contrast, cell-free modalities provide a more controlled and reproducible therapeutic input, with reduced dependence on viability constraints, but are inherently limited by finite bioactive content and lack the capacity for ongoing modulation in response to evolving microenvironmental conditions.

### 9.2. Interaction with the Disc Microenvironment

The intervertebral disc microenvironment represents a critical determinant of therapeutic efficacy, characterized by hypoxia, limited nutrient diffusion, acidic pH, and sustained mechanical loading. These conditions collectively impair cellular metabolism, reduce biosynthetic activity, and promote catabolic and inflammatory signaling within resident disc cells. For cell-based therapies, such constraints directly compromise cell viability, proliferative capacity, and functional activity following intradiscal administration. Reduced oxygen tension and glucose availability limit mitochondrial function and energy production, while acidic conditions disrupt intracellular homeostasis, leading to apoptosis and diminished extracellular matrix synthesis.

In contrast, cell-free approaches are not subject to viability constraints; however, their activity remains strongly influenced by the physicochemical properties of the disc environment. Bioactive molecules delivered in acellular formulations are susceptible to rapid diffusion away from the injection site, enzymatic degradation, and dilution within the extracellular matrix, resulting in limited residence time and reduced therapeutic exposure. In addition, the dense and avascular nature of the disc restricts the distribution of these agents, potentially limiting penetration into regions of advanced degeneration.

Furthermore, the biochemical characteristics of the disc microenvironment, including acidic pH, low glucose availability, and elevated enzymatic activity, may directly influence the stability and functional integrity of cell-free products. EVs and secretome-derived factors are susceptible to degradation or structural alteration under these conditions, which may affect the bioavailability and activity of their molecular cargo, including proteins and regulatory RNAs. As such, the duration and consistency of therapeutic effects may be limited by microenvironmental constraints, despite the absence of viability-dependent mechanisms.

Importantly, the challenges associated with delivery, retention, and sustained bioactivity are not exclusive to a single modality but represent shared translational barriers. Effective therapeutic strategies must therefore address both spatial localization and temporal persistence within the disc. Approaches such as biomaterial-based carriers, hydrogel systems, and controlled-release platforms have been developed to enhance retention, protect bioactive components from degradation, and enable sustained delivery. These strategies are applicable across both cell-based and cell-free therapies, highlighting the central role of the microenvironment in shaping therapeutic performance.

### 9.3. Clinical Maturity and Translational Status

Substantial heterogeneity exists in the degree of clinical translation across regenerative modalities, reflecting differences in developmental timelines, regulatory pathways, and underlying biological complexity. Cell-based therapies have advanced to late-phase clinical evaluation, including randomized controlled trials with placebo or active comparators. These studies have consistently demonstrated acceptable safety profiles; however, therapeutic efficacy has been variable, with several trials failing to meet primary endpoints. This discrepancy between preclinical promise and clinical outcomes highlights the challenges associated with translating biologically complex interventions into reproducible clinical benefit, particularly in the context of patient heterogeneity and multifactorial pain etiology.

PRP is supported by a broader clinical literature encompassing randomized trials, prospective cohort studies, and systematic syntheses. While many studies report improvements in pain and functional outcomes, variability in study design, injection protocols, and product composition complicates interpretation and limits the ability to define standardized treatment paradigms. Consequently, the clinical signal associated with PRP is best understood at a population level rather than through individual trials, with evidence suggesting modest but inconsistent therapeutic benefit.

In contrast, emerging cell-free modalities, including extracellular vesicle-based therapies and MSC-derived secretomes, remain largely confined to preclinical investigation or early translational studies. The absence of randomized clinical trials in spinal applications precludes robust assessment of clinical efficacy and safety in human populations. As a result, comparisons across modalities must account for disparities not only in the quantity of available data but also in the level of clinical validation and regulatory progression.

### 9.4. Standardization, Manufacturing, and Scalability

The feasibility of standardization and large-scale manufacturing represents a critical determinant of clinical translation and widespread adoption. Cell-based therapies are inherently variable, with product characteristics influenced by cell source, donor variability, expansion conditions, passage number, and culture environment. These factors contribute to heterogeneity in cellular phenotype, secretory profile, and functional potency, complicating reproducibility across batches and clinical studies. In addition, stringent regulatory requirements for cell processing, including good manufacturing practice compliance, impose logistical and economic constraints on scalability.

PRP, although simpler to prepare, is similarly affected by variability in platelet concentration, leukocyte content, activation methods, and processing systems. This lack of uniformity results in substantial differences in growth factor composition and biological activity between preparations, limiting comparability across studies and hindering the establishment of standardized clinical protocols.

This variability is particularly important when interpreting PRP studies, as differences in platelet concentration, leukocyte content, activation method, anticoagulant use, centrifugation protocol, injection volume, and delivery target may produce biologically distinct products that are all labeled as PRP. Similar concerns apply to other biologic products, including MSCs, platelet lysate, EVs, and secretomes, where donor source, processing methods, culture conditions, isolation techniques, and potency assays vary across studies. As a result, findings from one product formulation or protocol should not be assumed to apply to another, and cross-study comparisons should be interpreted cautiously.

Cell-free approaches offer theoretical advantages in manufacturing control, including the potential for batch production, extended storage, and centralized quality assurance. However, these advantages remain incompletely realized in practice. In particular, these systems remain dependent on upstream large-scale cell expansion, followed by technically demanding isolation and purification processes that are associated with variable yield, product loss, and potential contamination risks. Challenges also persist in defining robust potency assays that correlate with therapeutic activity, ensuring consistency in molecular composition across production batches, and establishing standardized isolation and purification methodologies. Furthermore, variability in upstream cell culture conditions directly influences the composition of derived products, introducing an additional layer of complexity. As such, while cell-free therapies may be more amenable to standardization from a manufacturing perspective, scalability is not inherently advantageous compared to cell-based therapies and remains contingent upon the development of robust, reproducible production platforms capable of supporting clinical translation.

### 9.5. Translational and Regulatory Considerations

Translational progression from preclinical development to clinical implementation is influenced not only by biological efficacy but also by regulatory classification, manufacturing requirements, and clinical feasibility. MSC-based therapies are generally regulated as advanced biologic products, necessitating rigorous evaluation through investigational new drug pathways, adherence to good manufacturing practice standards, and demonstration of safety, consistency, and potency. These requirements, while essential for clinical translation, impose significant logistical and economic constraints that may limit scalability and widespread adoption.

PRP occupies a comparatively less restrictive regulatory space in many jurisdictions, often categorized as a minimally manipulated autologous product. This facilitates more rapid clinical implementation; however, the absence of standardized preparation protocols and variability in product composition complicate regulatory oversight and limit reproducibility across clinical settings. Furthermore, intradiscal application frequently falls outside approved indications, introducing additional considerations related to off-label use.

Cell-free modalities, including EV-based therapies and MSC-derived secretomes, remain within an evolving regulatory landscape. Uncertainty persists regarding their classification as biologics, drugs, or advanced therapy products, with corresponding implications for manufacturing standards, quality control, and clinical approval pathways. In addition, the lack of validated potency assays and standardized characterization criteria represents a critical barrier to regulatory acceptance. These factors collectively contribute to delayed clinical translation despite substantial preclinical interest.

### 9.6. Integrated Comparative Perspective

Integration of the preceding domains highlights that each regenerative modality is defined by a distinct balance of biological capability, clinical maturity, and translational feasibility. MSC-based therapies offer the potential for adaptive and sustained biological activity, but their clinical performance is constrained by challenges related to cell survival, delivery, and manufacturing complexity. PRP demonstrates broader clinical utilization and accessibility, supported by an extensive but heterogeneous literature, with therapeutic effects that are generally modest and variable.

Emerging cell-free approaches, including EV-based therapies and MSC-derived secretomes, present theoretical advantages in manufacturing control, scalability, and avoidance of viability-dependent limitations. However, these modalities remain limited by early-stage clinical development, unresolved standardization challenges, and an absence of robust clinical validation. Importantly, the hostile disc microenvironment, delivery constraints, and lack of standardized therapeutic platforms represent shared barriers across all modalities, irrespective of their mechanistic differences.

Collectively, these observations indicate that no single approach consistently demonstrates superiority across the domains relevant to clinical application. Instead, the comparative analysis highlights modality-specific strengths and limitations that must be considered in the context of disease stage, patient selection, and therapeutic objectives. Advancing the field will require not only optimization within individual modalities but also rigorously designed comparative clinical studies capable of defining their relative efficacy and appropriate clinical roles in the management of IVDD-associated CLBP.

In addition to the regenerative modalities discussed in this review, a growing number of emerging cell-free therapeutic strategies are being investigated for intervertebral disc degeneration. These include senolytic agents targeting the accumulation of senescent nucleus pulposus cells, which have been implicated in age-related disc degeneration and inflammatory signaling. Similarly, molecular approaches aimed at modulating key inflammatory pathways, such as NF-κB decoy oligodeoxynucleotides, have demonstrated potential in preclinical models by attenuating pro-inflammatory cytokine expression and matrix degradation. Peptide-based therapies, including bioactive peptides designed to promote extracellular matrix synthesis or inhibit catabolic signaling, also represent a developing area of interest. While these strategies show promise, most remain at early preclinical or translational stages, with limited clinical validation in spinal applications. Accordingly, they are not included in the primary comparative framework of this review, which focuses on regenerative modalities with more advanced translational and clinical evidence, including platelet-derived products and MSC-based cell-free therapies.

## 10. Disc-Specific Translational Challenges

### 10.1. Delivery and Retention

Intradiscal injection is the standard delivery route, but retention of injected material is a significant concern. The NP is under compressive load, and injected fluids may leak through AF tears or the needle tract [59]. For cell-based therapies, this leakage reduces the delivered dose. For cell-free therapies, rapid clearance through endplate diffusion may limit exposure time. Biomaterial carriers, including hydrogels (thermosensitive, photo-crosslinkable), microspheres, and injectable scaffolds, are being developed to improve retention and provide sustained release of bioactive factors [43,100]. For EVs specifically, loading into hyaluronic acid-based hydrogels, gelatin methacrylate scaffolds, or self-assembling peptide hydrogels has improved disc retention and sustained therapeutic effect in preclinical models [99,111].

### 10.2. Potency Assays and Quality Control

No validated, disc-specific potency assay exists for any regenerative modality. For PRP, platelet concentration and growth factor profiles are commonly measured but do not predict clinical outcomes [87]. For MSC products, colony-forming unit assays, surface marker panels (CD73+/CD90+/CD105+/CD34−/CD45−), and trilineage differentiation capacity are standard but not specific to disc regeneration capacity [37]. For EVs, nanoparticle tracking analysis (NTA), surface marker expression (CD9, CD63, CD81), and proteomic/miRNA profiling provide characterization but not functional potency measures [102,103]. Development of functional assays, for example, NP cell matrix production in response to the therapeutic product, or suppression of IL-1β-induced catabolism, would be a significant advance for the field [63].

### 10.3. Patient Selection and Phenotyping

The RESPINE and Mesoblast trials highlight the critical importance of patient selection. Not all CLBP cases are discogenic, and not all disc degeneration is symptomatic. The Mesoblast trial found enhanced efficacy in patients with shorter LBP duration (<68 months), suggesting that earlier-stage disease may be more amenable to biological intervention [73]. Pfirrmann MRI grading (III–IV, moderate degeneration) may define a therapeutic window where sufficient cellular substrate remains for regenerative responses, whereas end-stage degeneration (grade V) may be beyond rescue [81,112]. Provocative discography, though controversial, and advanced imaging biomarkers (quantitative T2 mapping, SMART [Spatial Mapping of Altered Resolution Times] MRI, sodium MRI for proteoglycan content) may help identify patients most likely to benefit [113].

Future trials should apply more rigorous and standardized phenotyping strategies to enrich for patients with true IVDD-associated discogenic pain. Potential inclusion criteria may include chronic axial low back pain refractory to conservative treatment, MRI evidence of moderate degeneration such as Pfirrmann grade III–IV, preserved disc height, high-intensity zones, Modic endplate changes where appropriate, and concordant pain reproduction on provocative discography when ethically and clinically justified. Conversely, exclusion criteria should carefully address competing pain generators, including facet arthropathy, sacroiliac joint dysfunction, symptomatic spinal stenosis, radiculopathy, instability, prior fusion at the target level, systemic inflammatory disease, active infection, and severe psychosocial confounders that may influence pain reporting or treatment response. Clinical trials should also prespecify responder definitions using validated minimum clinically important differences for pain and function, such as VAS/NRS and ODI thresholds, to distinguish statistically significant changes from clinically meaningful improvement.

A major limitation across clinical studies is the lack of a standardized and validated approach to confirming discogenic pain. Many trials rely on imaging findings, such as MRI-defined degeneration, in combination with clinical symptoms, without definitive confirmation of the disc as the primary pain generator. This introduces heterogeneity in study populations and may confound interpretation of therapeutic efficacy, particularly in trials that include patients with multifactorial sources of low back pain.

### 10.4. Trial Design Considerations

Beyond patient phenotyping, several design features influence interpretation of regenerative therapy trials for IVDD-associated CLBP. Sham responses can be substantial in interventional pain studies, particularly when procedures involve image-guided intradiscal injection. Accordingly, adequately powered sham-controlled or active-comparator trials are needed to distinguish treatment-specific effects from contextual or procedural responses.

Endpoint selection also remains challenging. Pain and disability scales such as VAS/NRS and ODI capture clinically meaningful symptoms but may be influenced by coexisting pain generators and psychosocial factors. Conversely, imaging outcomes such as disc height, MRI signal intensity, or quantitative MRI biomarkers may suggest structural change but do not always correlate with symptom improvement. Future trials should therefore prespecify primary endpoints, responder definitions, and minimum clinically important difference thresholds for pain and function. Structural outcomes should be analyzed separately from analgesic outcomes to avoid equating pain improvement with biological disc regeneration.

Finally, secondary and subgroup findings should be interpreted cautiously when primary endpoints are not met. Unless these analyses are prespecified and adjusted for multiplicity, they should be considered exploratory and hypothesis-generating rather than confirmatory evidence of efficacy.

## 11. Combination and Multi-Target Strategies

Given the limitations of individual modalities, combination approaches are receiving increasing attention. MSCs combined with PRP or PL may benefit from the growth factors providing a supportive microenvironment that enhances transplanted cell survival and paracrine activity [33]. For the disc specifically, an MSC+PRP combination has shown enhanced NP cell proliferation and matrix production compared to either alone in porcine models [84].

Multi-target injection strategies addressing the disc, facet joints, and paraspinal muscles in a single session have primarily been explored with PRP. One large case series reported a 71% overall success rate at one year with weekly PRP injections into lower back muscles combined with physiotherapy, with post-procedure MRI showing improvement in pre-existing multifidus atrophy [40,86]. This approach acknowledges the multifactorial nature of CLBP and may be more effective than targeting any single structure in isolation.

Engineered EVs represent a further frontier. Loading EVs with specific therapeutic miRNAs (e.g., miR-142-3p for anti-apoptotic effects, or anti-miR-221 for matrix protection) or proteins, and functionalizing their surfaces with targeting peptides for NP cell uptake, may create precision therapeutics tailored to specific degenerative phenotypes [44,100]. While still preclinical, these approaches offer a path toward rational, mechanism-based therapy design. It should be noted that the relative strength of preclinical evidence does not necessarily correspond to the level of clinical validation, which remains more advanced for certain modalities such as PRP.

## 12. Safety Considerations Across Regenerative Modalities

Safety remains a central consideration for intradiscal and perispinal regenerative therapies (Table 2). Although many clinical studies report acceptable short-term safety profiles, the risks differ across modalities and are influenced by product composition, injection target, delivery technique, and patient selection. Procedure-related risks common to intradiscal interventions include discitis, post-injection pain flare, bleeding, nerve irritation, leakage through annular defects, and potential acceleration of disc degeneration related to needle puncture or repeated procedures. These risks are particularly relevant because the intervertebral disc is avascular, has limited immune surveillance, and may be vulnerable to additional structural disruption.

For MSC-based therapies, additional theoretical risks include poor cell survival within the hostile disc microenvironment, immune reactions to allogeneic products, ectopic tissue formation, and osteophyte formation following cell leakage outside the disc space. Platelet-derived products such as PRP and platelet lysate are generally considered lower risk when autologous or appropriately screened; however, they may still cause transient inflammatory flares, pain exacerbation, infection, or variability-related safety concerns depending on leukocyte content, activation method, and preparation protocol. Allogeneic platelet lysate may also carry theoretical immunologic or donor-related risks despite processing and screening.

For extracellular vesicle and secretome-based therapies, clinical safety remains insufficiently defined because spinal applications are largely preclinical. Key uncertainties include biodistribution after intradiscal injection, persistence within the disc space, off-target signaling, immunogenicity, dose–response relationships, and long-term effects on resident disc cells. As EVs and secretomes contain complex bioactive cargo, including proteins, lipids, and regulatory nucleic acids, rigorous characterization, sterility testing, potency assays, and long-term surveillance will be required before clinical translation.

Overall, available evidence suggests that regenerative therapies for IVDD-associated CLBP are generally feasible, but definitive conclusions regarding comparative safety cannot yet be drawn. Future trials should incorporate standardized adverse event reporting, longer follow-up, imaging-based assessment of structural changes, and explicit monitoring for infection, accelerated degeneration, ectopic tissue formation, immune reactions, and procedure-related complications.

## 13. Regulatory and Manufacturing Considerations

The regulatory classification of regenerative products varies by jurisdiction and profoundly affects the path to clinical adoption. In the United States, autologous PRP prepared at point of care is generally regulated as a minimally manipulated autologous tissue product under 21 CFR Part 1271 and does not require premarket approval. However, intradiscal use is considered off-label for most FDA-cleared PRP preparation systems. MSC products are classified as somatic cell therapies (biologics) requiring Investigational New Drug (IND) applications and formal clinical trial pathways [37]. EVs and secretomes occupy an evolving regulatory space: the FDA has issued guidance treating therapeutic exosome products as biological drugs requiring full biologics licensing, but enforcement and specific manufacturing requirements continue to develop [102].

In the European Union, MSC products fall under the Advanced Therapy Medicinal Products (ATMP) regulation (EC No 1394/2007), which imposes stringent requirements for GMP-compliant manufacturing, traceability, and pharmacovigilance [114,115,116]. PRP regulation varies by member state, creating an inconsistent landscape. Exosome products are likely to be classified as ATMPs or medicinal products depending on their composition and claims.

From a manufacturing perspective, PRP is the simplest to produce but the hardest to standardize. MSC expansion requires GMP-compliant cell processing facilities; and exosome/secretome production requires both cell culture and downstream purification infrastructure. Platelet lysate occupies a middle ground: it can be produced from banked platelet concentrates under blood banking GMP standards and may leverage existing transfusion medicine infrastructure for scalable production [89,92].

## 14. Limitations

Several limitations should be acknowledged. First, this is a narrative rather than systematic review, and although a structured search strategy was used, study selection may be subject to selection bias. Second, direct comparative studies between the reviewed modalities are lacking, limiting conclusions regarding relative efficacy. Third, substantial heterogeneity in product preparation, delivery protocols, patient selection, and outcome measures limits cross-study comparison. Fourth, most evidence for EV- and secretome-based therapies remains preclinical, and translation to human IVDD is uncertain. Fifth, the definition of discogenic pain varies across clinical studies and is often based on imaging and clinical correlation rather than definitive diagnostic confirmation. Finally, cost-effectiveness data and long-term safety data remain limited for all modalities in spinal applications.

## 15. Conclusions and Future Directions

The regenerative medicine landscape for CLBP secondary to IVDD has evolved substantially over the past decade, while also revealing important translational limitations. Intradiscal MSC therapy, once regarded as the most promising biologic strategy, has consistently shown acceptable safety profiles but has not produced convincing efficacy signals in rigorously designed, placebo-controlled clinical trials. In contrast, PRP currently possesses the most extensive clinical evidence base and the most feasible pathway for clinical implementation. Nevertheless, heterogeneity in preparation protocols, incomplete mechanistic understanding, and lack of standardization across studies continue to limit confidence in its capacity to induce true structural regeneration.

Cell-free biologic approaches, including platelet lysate, MSC-derived EVs, and MSC-derived secretomes, offer several theoretical advantages relative to cell-based therapies. These include improved manufacturing scalability, greater potential for standardized batch production, and avoidance of the cell survival challenges imposed by the hypoxic, acidic, and nutrient-limited disc microenvironment. Despite these conceptual advantages, such approaches remain largely confined to preclinical investigation or early translational development, with no completed randomized clinical trials evaluating their efficacy for IVDD-associated CLBP.

Several priorities are critical for advancing the field. First, standardized preparation, characterization, and reporting protocols are required across all regenerative modalities; without methodological consistency, clinical trials will continue to yield heterogeneous and non-comparable results. Second, development of disc-specific delivery systems, such as injectable hydrogels, microspheres, and biomaterial carriers, is essential to improve retention and enable sustained therapeutic activity within the mechanically loaded, avascular disc environment. Third, improved patient phenotyping strategies are needed to identify individuals whose CLBP is predominantly discogenic and whose stage of degeneration remains amenable to biological intervention. Such strategies may incorporate advanced imaging biomarkers, provocative diagnostic testing, and emerging molecular markers of disc pathology. Fourth, rigorously designed comparative clinical trials are necessary, ideally incorporating adaptive or platform trial designs that allow direct evaluation of multiple biologic modalities rather than the current landscape of isolated single-intervention studies.

This review provides a framework for evaluating regenerative therapies within a broader translational context rather than as isolated modalities. At present, no biological intervention has demonstrated definitive regenerative efficacy for CLBP secondary to IVDD, and clinicians should exercise caution regarding the premature adoption of commercially marketed regenerative products outside well-designed clinical trials. By directly comparing cell-based and cell-free regenerative strategies within a single framework, this review highlights areas of convergence, divergence, and unmet research needs that may not be apparent when these therapies are evaluated separately. Future progress will require coordinated multidisciplinary collaboration integrating spine surgery, pain medicine, cell biology, biomaterials engineering, and regulatory science.

## Figures and Tables

**Table 1:** Comparative overview of regenerative modalities for chronic low back pain secondary to intervertebral disc degeneration.

Parameter	Intradiscal MSC	PRP	Platelet Lysate	MSC EVs	MSC Secretome
Source	Autologous or allogeneic (BM, adipose, UC)	Autologous whole blood	Allogeneic or autologous platelet concentrates	Allogeneic MSC culture supernatant	Allogeneic MSC conditioned medium
Contains viable nucleated cells	Yes	No	No	No	No
Principal bioactive components	Viable MSCs and paracrine mediators	Platelets, growth factors, cytokines, and variable leukocyte content	Pre-released platelet growth factors, cytokines, and possible EV components	Membrane-bound vesicles containing proteins, lipids, and nucleic acids	Soluble proteins, cytokines, growth factors, metabolites, and EV components
Highest level of clinical evidence for IVDD- associated CLBP	Phase III RCT (failed primary endpoint)	Multiple clinical studies, but heterogeneous by injection target and protocol	No RCT for spinal application	No human trials for IVDD	No human trials for IVDD
Standardization	Low (cell source, dose, passage vary)	Low (preparation method dependent)	Moderate to high (batch production feasible)	Low (isolation method dependent)	Low to moderate (culture conditions vary)
Immunogenicity considerations	Low to moderate; greater concern for allogeneic products	Generally low when autologous	Low to uncertain; donor-derived products require screening	Uncertain; dependent on source, purification, and cargo	Uncertain; dependent on source, composition, and manufacturing
Manufacturing/scalability considerations	Limited by donor variability, cell expansion, and GMP processing	Point-of-care feasible but poorly standardized	Batch production feasible, but standardization required	Technically challenging; dependent on cell expansion and EV isolation	Technically challenging; dependent on cell culture and product characterization
Regulatory considerations	Advanced biologic or cell therapy; jurisdiction-dependent	Often treated as minimally manipulated autologous product, but varies by use and jurisdiction	Blood-derived biologic or medicinal product; depends on source, manipulation, and jurisdiction	Evolving classification; likely biologic or drug depending on claims and manufacturing	Evolving classification; likely biologic or drug depending on composition and claims

Abbreviations: BM, bone marrow; UC, umbilical cord; EV, extracellular vesicle; RCT, randomized controlled trial; IVDD, intervertebral disc degeneration.

**Table 2:** Safety considerations for regenerative therapies for IVDD-associated chronic low back pain.

Modality	Procedure-Related Risks	Product-Specific Risks	Key Unresolved Safety Issues
MSC-based therapy	Discitis, post-injection flare, bleeding, nerve irritation, annular leakage, degeneration related to needle puncture or repeated intradiscal procedures	Immune reaction to allogeneic cells, poor cell survival, ectopic tissue formation, osteophyte formation after leakage	Long-term persistence, optimal dose, effects of repeated injections, risk in advanced annular disruption
PRP	Discitis, transient pain flare, bleeding, nerve irritation, leakage through annular defects	Variability in leukocyte content, inflammatory response, activation-dependent effects	Whether repeated injections affect disc integrity; unclear relationship between PRP composition and adverse events
Platelet lysate	Similar injection-related risks to PRP if delivered intradiscally	Donor-related or immunologic risk for allogeneic products; variability in growth factor and EV content	Long-term safety of standardized allogeneic preparations in the disc environment
MSC-derived EVs	Risks related to intradiscal injection, including discitis, flare, leakage, and needle-related degeneration	Off-target signaling, uncertain immunogenicity, cargo variability	Biodistribution, persistence, dose–response, long-term effects on resident disc cells
MSC-derived secretome	Similar intradiscal injection-related risks	Complex and variable bioactive cargo; potential off-target biological effects	Biodistribution, stability, potency, sterility, and long-term safety remain undefined

## Data Availability

No new data were created or analyzed in this study.

## Abbreviations

AF	Annulus fibrosus
AD-MSCs	Adipose-derived mesenchymal stromal cells
BM-MSCs	Bone marrow-derived mesenchymal stromal cells
CEPs	Cartilaginous endplates
CLBP	Chronic low back pain
CM	Conditioned media
ECM	Extracellular matrix
EVs	Extracellular vesicles
HA	Hyaluronic acid
IVDD	Intervertebral disc degeneration
LBP	Low back pain
MMPs	Matrix metalloproteinases
MSCs	Mesenchymal stromal cells
NP	Nucleus pulposus
ODI	Oswestry Disability Index
PL	Platelet lysate
PRP	Platelet-rich plasma
RCT	Randomized controlled trial
TNF-α	Tumor necrosis factor alpha
UC-MSCs	Umbilical cord-derived mesenchymal stromal cells
VAS	Visual Analog Scale
VEGF	Vascular endothelial growth factor
